# Impact of standardized tobacco packaging on smoking-related behaviors and perceptions in Singapore

**DOI:** 10.18332/tid/189551

**Published:** 2024-08-01

**Authors:** Lionel Ng, Chuen Seng Tan, Jeong Kyu Lee, Yvette van der Eijk

**Affiliations:** 1 Saw Swee Hock School of Public Health, National University of Singapore and National University Health System, Singapore, Singapore; 2 Department of Health and Exercise Science, The University of Oklahoma, Norman, United States

**Keywords:** tobacco marketing, plain packaging, tobacco policy, tobacco packaging, adult smokers

## Abstract

**INTRODUCTION:**

Singapore phased in standardized tobacco packaging on 1 July 2020 following a three-month grace period. This pre-post study evaluated its impacts on smoking-related behaviors and perceptions among adults who currently smoke.

**METHODS:**

Baseline and follow-up data were collected in a pre- and post-questionnaire from a cohort of 1873 Singaporean adults who were currently smoking at baseline. Baseline data were collected from December 2019 to May 2020, and follow-up data from July 2021 to September 2021. We used descriptive statistics and bivariate analyses to assess pre-post changes (Bhapkar’s test, Wilcoxon signed rank test) and to identify characteristics of participants who had quit or cut down smoking at follow-up (Pearson’s chi-squared, Fisher’s exact test).

**RESULTS:**

At follow-up, 11.7% (n=220) had quit smoking. There was a higher proportion of those smoking non-daily (pre: 13.1%, post: 16.9%; p<0.001), and those intending to quit within the next year (pre: 14.8%, post: 17.5%; p<0.05) or six months (pre: 10.4%, post: 13.2%; p<0.01). Tobacco products were scored more negatively in relation to packaging, quality, satisfaction, value for money and overall appeal (scores pre: 15.9, post: 14.3; p<0.001), harmfulness (scores pre: 0.61, post: 0.54; p<0.05), noticing others smoking the same brand (scores pre: 1.92, post: 1.65; p<0.001), and considering quitting due to health warnings (scores pre: 0.81, post: 0.86, p<0.05). Fewer reported that some cigarette brands have higher prestige (pre: 58.0, post: 54.3%; p<0.01), and more reported using flavored cigarettes (pre: 42.2%, post: 60.1%; p<0.001) and e-cigarettes (pre: 4.2%, post: 6.1%; p<0.01).

**CONCLUSIONS:**

In Singapore, the changes observed before and after the implementation of standardized packaging suggest that it might be associated with quit-related outcomes, reduced tobacco product appeal, and increased effectiveness of graphic health warnings.

## INTRODUCTION

Standardized tobacco packaging strips all colors, logos and branding elements from tobacco packs. Since Australia first introduced standardized packaging in 2012, a number of other countries in Europe, Australasia, USA, Asia and the Middle East have followed suit with laws that standardize pack size, color and text, remove all logos and branding, and require graphic health warnings to be printed on tobacco packs in standardized formats^1^. Singapore was the second Southeast Asian country after Thailand to introduce standardized packaging on 1 July 2020, following a three-month grace period from 1 April to 30 June 2020^2^.

Singapore’s standardized packaging mandate requires all tobacco products to be packaged in matte finished, dark brown colored packs of a standard size, with six rotating graphic health warnings covering at least 75% of the pack, and brand and variant names shown in a standardized font, size, length and position on the pack. The mandate also standardizes the pack texture, opening mechanism (only flip top is permitted) and cigarette stick dimensions, and does not permit decorative or other marketing features on the pack, cellophane wrapping or cigarette sticks, with the stick filters restricted to either plain cork or white colors^3^.

Evaluation studies of similar standardized packaging policies have been done in Australia, the United Kingdom, France, Norway and Canada, in longitudinal cohorts and serial cross-sectional samples to assess quit-related behaviors, health warning salience, cognitive reactions and tobacco-related perceptions. These suggest that standardized packaging increases the salience of graphic health warnings on packs^4-8^ and increases awareness on the harms of smoking^4,5,8-10^. The studies also reported a range of behavioral changes in people currently smoking including avoiding the warning labels, concealing the packs, requesting for packs with different health warnings, and feelings of self-consciousness when taking out the cigarette pack^4,5,7,8,11,12^. Studies also reported reduced tobacco product appeal and more negative perceptions of cigarette packs among people who smoke^5-7,9,13,14^, a reduced likelihood of smoking among adults and youths^5,7-9,11,15-19^, and increased support for standardized packaging regulations following their implementation among people who smoke^9,13,20^. However, fewer studies to evaluate plain packaging have been done in the other regions, notably the countries in Asia, Latin America, and the Middle East.

This pre-post study aimed to evaluate the impacts of Singapore standardized tobacco packaging, specifically on smoking-related behaviors and perceptions among adults who smoke.

## METHODS

### Study design and participants

We collected pre- and post-intervention data from the Singapore Smokers Survey (SSS) cohort. An *a priori* power analysis based on results of a prior study^7^ and conservative power threshold of 0.95 revealed that a sample size of 2140 was required. We recruited a slightly larger sample at baseline (n=2279) to account for dropouts and non-responses at follow-up. Pre-intervention (baseline) data were collected from 3 December 2019 to 2 May 2020, at least two months prior to standardized packaging implementation. Of the 2279 participants in the baseline survey, 338 (14.8%) completed the survey during the standardized packaging phase-in period (from 1 April to 2 May 2020). Post-intervention (follow-up) data were collected from the same participants from 1 July to 5 September 2021, at least 12 months after standardized packaging implementation. Of the 2279 baseline survey participants, 86 refused to be re-contacted for future research. Among the 2193 participants who agreed to be recontacted, 1873 completed the follow-up survey with a response rate of 85.4%.

Participants met the eligibility criteria if they were adults (aged ≥18 years at baseline), Singapore Citizens/Permanent Residents, and currently smoking at baseline. Following the Centers for Disease Control and Prevention definition^21^, we defined ‘current smokers’ as those who have smoked at least 100 cigarettes in their lifetime and were smoking cigarettes on a daily or non-daily basis at the time of survey. In the follow-up survey, we also included participants who had quit smoking since the baseline survey.

### Procedures

Participants were recruited via convenience methods into the baseline SSS from existing cohort studies ran by the Singapore Population Health Studies (SPHS) by selecting participants who, in prior studies, had reported current smoking. These cohorts included the SPHS cohort first follow-up study, Multi-Ethnic Cohort Phase 3 study, SPHS Online Panel, and 2016–2017 National Population Health Survey^22^, with further convenience recruitment in designated smoking areas in public places such as streets, near coffee shops, bars, and restaurants, through personal contacts (e.g. through existing social networks and word of mouth), and distribution of recruitment flyers. For the follow-up SSS, an invitation was sent to all 2193 participants who had agreed to be recontacted. Those who did not respond to the invitation within two weeks were contacted again by a telephone call by a staff member of the SPHS operations team.

Data were provided in a self-administered online questionnaire or interviewer-administered telephone questionnaire, with questions on sociodemographics, smoking-related behaviors, and perceptions of tobacco products. At baseline, 185 participants completed the survey over the phone and 2094 completed the survey online. Each participant was reimbursed with $10 Singapore Dollars for completing the baseline survey, and an additional $20 Singapore Dollars for completing the follow-up survey. The surveys and supporting documents were available in all four national languages (English, Chinese, Malay and Tamil).

### Measurements

*Sociodemographics*

We collected data on participants’ age, gender, ethnicity (Chinese, Malay, Indian, others), and monthly household income in SGD (<2000, 2000–5999, 6000–9999, ≥10000) (1000 Singapore dollars about US$740).

*Smoking-related behaviors*

We categorized participants as daily, non-daily, or former smokers based on a question which asked whether they were smoking cigarettes or any other tobacco products ‘daily’ (daily smoker), ‘at least weekly’ or ‘less often than weekly’ (non-daily smoker) or ‘not at all’ (former smoker) at the time of survey. To enable a distinction of daily smokers based on their nicotine dependence level, we used the Heaviness of Smoking Index (HSI)^23^. HSI was calculated using two questions: ‘On days that you smoke, how soon after you wake up do you have your first cigarette?’ with four response options (after 60 min, within 31–60, within 6–30, and within 5 min) and ‘On days that you smoke, how many cigarettes do you typically smoke per day?’ with four response options (≤10, 11–20, 21–30, ≥31). Each response option was progressively assigned a score ranging 0–3. Participants were then categorized as having low dependence (HSI score 0–1), moderate dependence (HSI score 2–4), or high dependence (HSI score 5–6). In our analysis, we combined those with moderate and high dependence due to low numbers. We collected data on intentions to quit (within next 30 days, within next 6 months, within next year, more than a year later, no intention to quit), and attempts to quit (for at least 24 hours in the past 12 months). We categorized ‘hardcore smokers’ as those consuming at least 15 cigarettes per day, with no quit intention or past 12-month quit attempt.

We assessed age of smoking initiation by asking at which age they smoked their first whole cigarette, and included questions on current e-cigarette use and preferred tobacco flavor. In the baseline survey, participants were asked open questions about their regular brand and variants, and flavors were coded manually against a database of tobacco variants on the Singapore market from a prior study^3^. In the follow-up survey, this question was simplified by asking whether their preferred cigarettes contained added flavor and, if so, to select the flavors from a list. In our analysis, we classified ‘flavored cigarettes’ as those including any additive or flavor that produces a noticeable smell or taste other than tobacco (e.g. menthol, fruits, sweets, clove).

*Perceptions of tobacco products*

We assessed perceived product appeal, perceived brand distinction, and the salience of health warnings of the tobacco products that participants were currently using with 16 questions, 13 of which were graded on a five-point Likert scale (Table 1). Likert scale questions were treated as continuous variables, with answers scored on a scale of one (most negative perception) to five (most positive perception). The three questions that did not use a five-point Likert scale (‘In your opinion, do you think that some cigarette brands have more prestige (higher status) than others?’, ‘When you see a pack of tobacco products that you usually buy or use, what do you usually notice first?’, ‘In the past month, have you asked for a pack of a tobacco product with a different health warning on it?’), were treated as categorical variables. In addition, we added up scores from the five questions assessing perceived product appeal (Table 1) to produce an overall product appeal score, graded on a scale of one (most negative perception) to 25 (most positive perception). The ‘product appeal’ construct was found to have good internal consistency (Cronbach’s alpha =0.76).

**Table 1:** Questions used to assess perceived product appeal, perceived brand distinction, and salience of health warnings in the baseline (2019–2020) and follow-up (2021) questionnaires to evaluate the impact of standardized packaging in Singapore

*Questions to assess perceived product appeal*
1. Do you like the packaging of the tobacco products (cigarettes and other types of tobacco products) that you usually buy or use?^a^
2. How do you rate the tobacco products you currently use in terms of quality?^a^
3. How do you rate the tobacco products you currently use in terms of satisfaction?^a^
4. How do you rate the tobacco products you currently use in terms of value for money?^a^
5. How do you rate the tobacco products you currently use in terms of appeal of the packaging?^a^
***Questions related to other product attributes***
1. In your opinion, do you think that some cigarette brands have more prestige (higher status) than others?^b^
2. In your opinion, do you think that different cigarette brands taste different from each other?^a^
3. In your opinion, how different in strength are the varieties within a cigarette brand?^a^
4. In your opinion, are some cigarette brands more harmful than others? ^a^
5. Do you sometimes find it hard to believe that the cigarette brand you are using is harmful to your health?^a^
6. In the past month, how often have you noticed other people smoking the same brand of cigarettes as you?^a^
7. Do you feel a connection with people who smoke the same brand as you?^a^
8. When you see a pack of tobacco products that you usually buy or use, what do you usually notice first?^b^
9. In the past month, have you asked for a pack of a tobacco product with a different health warning on it?^b^
10. In the last one month, how often did you purposely cover up or conceal your pack of tobacco product(s), or put your tobacco product(s) in another container?^a^
11. In the last one month, to what extent – if at all – have the health warnings on the packs of tobacco products made you consider quitting smoking? ^a^

a Responses scored on a five-point Likert scale and treated as continuous variables in our analysis.

b Responses treated as categorical variables in our analysis.

### Data analysis

We used descriptive statistics to summarize the sociodemographics and smoking-related behaviors of participants, with frequencies and percentages reported for categorical variables, and mean and standard deviation for continuous variables. We used bivariate analyses to assess pre-post changes in smoking-related behaviors and perceptions of tobacco products. We used Bhapkar’s test for categorical variables, followed with McNemar’s test if the difference was significant and the variable had three or more groups to determine which group(s) contributed to the significant difference. As all continuous variables were found to have non-normal distributions (w=0.61–0.98, p<0.001, Shapiro-Wilk) we used Wilcoxon signed rank test to assess changes in pre and post results. We conducted an additional bivariate analysis to compare the characteristics of participants who had quit smoking, cut down cigarette consumption, or increased/not changed cigarette consumption from baseline to follow-up, and tested strength of association with Pearson’s chi-squared test (n>5) or Fisher’s exact test (n≤5). If the difference was significant and the variables had three or more groups, we used standardized residuals of chi-squared to determine which group contributed to the significant difference. All tests were two-tailed and we set all significance thresholds at p<0.05 and accounted for Type 1 error by also reporting p values at the more conservative thresholds of p<0.01 and p<0.001. We conducted all analyses in RStudio V.2023.03.0.

## RESULTS

### Sociodemographics and smoking-related behaviors

At follow-up, the majority of participants were aged 25–44 years with a mean age of 39.6 years (SD=12.6), male, of Chinese ethnicity, and of middle income (Table 2). In all, 23.0% (n=262) of participants were characterized as ‘hardcore smokers’: those who smoke at least 15 cigarettes per day and report no past-12 month attempts or intention to quit. The mean age of smoking initiation was 15.9 years (SD=3.7), and 64.4% (n=1053) had initiated smoking before the age of 18 years. Among those who were smoking daily at follow-up, 54.7% (n=706) reported intentions to quit although only 4.1% (n=53) intended to quit within the next 30 days, and 36.3% (n=469) had attempted to quit in the past 12 months; 60.1% (n=897) used flavored cigarettes, and 6.1% (n=114) reported current use of e-cigarettes.

**Table 2:** Summary of sample sociodemographic characteristics and smoking-related behaviors in the 2021 follow-up survey of the Singapore Smokers Survey cohort (N=1873)

*Characteristics*	*n (%)*
**Age** (years)	
18–24	215 (11.5)
25–44	1011 (54.0)
45–64	591 (31.6)
≥65	56 (3.0)
**Gender**	
Male	1420 (75.8)
Female	453 (24.2)
**Ethnicity**	
Chinese	1177 (62.8)
Malay	405 (21.6)
Indian	256 (13.7)
Other	35 (1.9)
**Monthly household income** (SGD) (N=1684)	
<2000	286 (17.0)
2000–5999	853 (50.7)
6000–10000	343 (20.4)
>10000	202 (12.0)
**Smoking status and dependence level** (N=1502)	
Daily smoker, moderate/high dependence	774 (51.5)
Daily smoker, low dependence	365 (24.3)
Non-daily smoker	232 (15.4)
Former smoker	131 (8.8)
**Hardcore smoking^a^** (N=1139)	
Hardcore smoker	262 (23.0)
Non-hardcore smoker	877 (77.0)
**Age of smoking initiation** (years) (N=1634)	
≤11	129 (7.9)
12–14	458 (28.0)
15–17	466 (28.5)
18–20	448 (27.4)
≥21	133 (8.1)
**Intention to quit smoking^a^** (N=1291)	
Within the next 30 days	53 (4.1)
Within the next 6 months	132 (10.2)
Within the next 1 year	204 (15.8)
More than a year later	317 (24.6)
No intention to quit	585 (45.3)
**Quit for 24 hours in past 12 months^a^** (N=1291)	
Yes	469 (36.3)
No	822 (63.7)
**Flavor of regular brand** (N=1492)	
Non-flavored	595 (39.9)
Flavored	897 (60.1)
**Current e-cigarette user**	
Yes	114 (6.1)
No	1759 (93.9)

a Daily smokers only. SGD: 1000 Singapore dollars about US$740.

At follow-up, 11.7% (n=220) of participants had quit smoking. Although there was a decrease in the proportion who had made a quit attempt (pre: 52.9%, post: 45.6%; p<0.001, χ^2^=12.7), there was an increase in the proportion of those smoking non-daily (pre: 13.3%, post: 16.9%; p<0.001, χ^2^=23.2) and decrease in the proportion of those smoking daily with moderate or high dependence (pre: 57.1%, post: 56.5%; p<0.05, χ^2^=3.9). While there was no significant change in the proportion of ‘hardcore smokers’, at follow-up there was an increase in the proportion intending to quit within the next year (pre: 14.8%, post: 17.5%; p<0.05, χ^2^=5.4) and next six months (pre: 10.4%, post: 13.2%; p<0.01, χ^2^=12.09), and a decrease in the proportion with no intention to quit (pre: 43.0%, post: 39.5%; p<0.001, χ^2^=23.02). At follow-up, a higher proportion were also using flavored cigarettes (pre: 42.2%, post: 60.1%; p<0.001, χ^2^=190.0), and e-cigarettes (pre: 4.2%, post: 6.1%; p<0.01, χ^2^=8.85) (Table 3).

**Table 3:** Comparison of smoking-related behaviors at baseline (2019–2020) and follow-up (2021) in the Singapore Smokers Survey cohort, pre- and post-implementation of the standardized tobacco packaging policy in Singapore

*Smoking behavior*	*n (%)*			
	*Baseline*	*Follow-up*	*p ^a^*	*χ^2^*
**Hardcore smoking**			0.328	1.0
Hardcore smoker	298 (22.3)	262 (23.0)		
Non-hardcore smoker	1036 (77.7)	877 (77.0)		
**Dependence level**			<0.001	24.8
Non-daily smoker	201 (13.1)***	232 (16.9)***		
Daily, low dependence	457 (29.8)	365 (26.6)		
Daily, moderate or high dependence	877 (57.1)*	774 (56.5)*		
**Quit attempt in past 12 months**			<0.001	12.7
Yes	991 (52.9)***	753 (45.6)***		
No	882 (47.1)***	900 (54.4)***		
**Quit smoking intention**			<0.001	39.6
Within the next 30 days	171 (9.1)	116 (7.0)		
Within the next 6 months	195 (10.4)**	219 (13.2)**		
Within the next 1 year	278 (14.8)*	290 (17.5)*		
More than a year later	423 (22.6)	375 (22.7)		
No intention to quit	806 (43.0)***	653 (39.5)***		
**Flavor of regular brand**			<0.001	190.0
Non-flavored	814 (57.8)***	595 (39.9)***		
Flavored	595 (42.2)***	897 (60.1)***		
**Current use of e-cigarettes**			0.003	8.9
Yes	79 (4.2)**	114 (6.1)**		
No	1794 (95.8)**	1759 (93.9)**		

a McNemar’s test (3 or more categories) or Bhapkar’s test (2 categories).

Statistically significant difference between proportions of categories indicated by:

* p<0.05,

** p<0.01,

*** p<0.001.

There were significant associations between changes in smoking behavior and age, ethnicity, dependence level, quit intention, and hardcore smoking, but not gender, household income, flavor or e-cigarette use (Table 4). Those who were aged 18–24 years (p<0.01, χ^2^=18.9), smoking non-daily (p<0.001, χ^2^=118.0), intending to quit in the next 30 days or 6 months (p<0.001 and p<0.05 respectively; χ^2^=64.6), and not ‘hardcore smokers’ (p<0.01, χ^2^=12.2) were more likely to have quit at follow-up, and those smoking daily with moderate or high dependence (p<0.001, χ^2^=118.0), those with no intention of quitting (p<0.001, χ^2^=64.6), and ‘hardcore smokers’ (p<0.01, χ^2^=12.2) at baseline were less likely to have quit at follow-up. Those of Malay ethnicity (p<0.01, χ^2^=16.6) and smoking daily with moderate or high dependence (p<0.001, χ^2^=118.0) were more likely to have cut down consumption, but not quit, at follow-up.

**Table 4:** Characteristics of those who reported having quit smoking, cut down cigarette consumption, or increased/not changed consumption at follow-up (2021) compared at baseline (2019–2020), following the implementation of standardized tobacco packaging in Singapore

*Variable*	*n (%)*				
	*Quit*	*Cut down*	*Increased/no change*	*p*	*χ^2^*
**Age** (years)	N=131	N=131	N=1108	0.004	19.0
18–24	21 (16.0)**	8 (6.1)	73 (6.6)		
25–44	60 (45.8)	63 (48.1)	590 (53.2)		
45–64	43 (32.8)	54 (41.2)	408 (36.8)		
>64	7 (5.3)	6 (4.6)	37 (3.3)		
**Gender**	N=131	N=131	N=1108	0.379	1.9
Male	97 (74.0)	105 (80.2)	876 (79.1)		
Female	34 (26.0)	26 (19.8)	232 (20.9)		
**Ethnicity**	N=131	N=131	N=1108	0.002	16.6
Chinese	79 (60.3)	64 (48.9)	679 (61.3)		
Malay	24 (18.3)	48 (36.6)**	255 (23.0)		
Indian/Other	28 (21.4)	19 (14.5)	174 (15.7)		
**Household income** (SGD)	N=115	N=122	N=1007	0.082	11.2
<2000	20 (17.4)	29 (23.8)	187 (18.6)		
2000–5999	50 (43.5)	63 (51.6)	534 (53.0)		
6000–10000	24 (20.9)	18 (14.8)	184 (18.3)		
>10000	21 (18.3)	12 (9.8)	102 (10.1)		
**Flavor of regular brand**	N=102	N=109	N=899	0.114	4.3
Non-flavored	57 (55.9)	74 (67.9)	521 (58.0)		
Flavored	45 (44.1)	35 (32.1)	378 (42.0)		
**Dependence level**	N=131	N=131	N=1108	<0.001	118.0
Non-daily	38 (29.0)***	11 (8.4)	103 (9.3)***		
Daily, low dependence	43 (32.8)	0 (0.0)***	362 (32.7)***		
Daily, moderate or high dependence	50 (38.2)***	120 (91.6)***	643 (58.0)		
**Quit smoking intention**	N=131	N=131	N=1108	<0.001	64.6
Within the next 30 days	26 (19.8)***	9 (6.9)	56 (5.1)***		
Within the next 6 months	23 (17.6)*	20 (15.3)	93 (8.4)**		
Within the next 1 year	17 (13.0)	18 (13.7)	151 (13.6)		
More than a year later	27 (20.6)	28 (21.4)	254 (22.9)		
No intention to quit	38 (29.0)***	56 (42.7)	554 (50.0)**		
**Hardcore smoking**	N=93	N=120	N=1005	0.002	12.2
Hardcore smoker	9 (9.7)**	23 (19.2)	250 (24.9)*		
Non-hardcore smoker	84 (90.3)**	97 (80.8)	755 (75.1)*		
**Current e-cigarette use**	N=131	N=131	N=1108	0.120^a^	
Yes	4 (3.1)	8 (6.1)	30 (2.7)		
No	127 (96.9)	123 (93.9)	1078 (97.3)		

Statistically significant difference between proportions of categories is indicated by:

* p<0.05,

** p<0.01,

*** p<0.001, calculated with standardized residuals of chi-squared. Data were categorized using baseline data.

a Fisher’s exact test. SGD: 1000 Singapore dollars about US$740.

Those who had increased or not changed their cigarette consumption at follow-up were more likely to be smoking daily with low dependence (p<0.001, χ^2^=118.0), have no intention to quit (p<0.01, χ^2^=64.6), and ‘hardcore smokers’ (p<0.05, χ^2^=12.2), and less likely to be smoking non-daily (p<0.001, χ^2^=118.0), intending to quit in the next 30 days (p<0.001, χ^2^=64.6) or 6 months (p<0.01), and not ‘hardcore smokers’ (p<0.05, χ^2^=12.2) at baseline.

### Perceptions of tobacco products

At follow-up, perceived product appeal was more negative in relation to tobacco product packaging, quality, satisfaction, value for money and overall appeal, on all five individual measures (all at p<0.001, V=426738, 230963, 198550, 318587, 489051, respectively) and for the overall product appeal score which combined data from the five indicators (p<0.001, V=1003183) (Table 5). Perceived brand distinction had also decreased in relation to prestige, harmfulness, and noticing others smoking the same brand. A higher proportion of participants responded ‘no’ (pre: 22.7%, post: 29.0%; p<0.001, χ^2^=25.9) at follow-up when asked if some brands have higher prestige than others. Perceiving some brands as more harmful than others (scores pre: 0.61, post: 0.54; p<0.05, V=73209), and noticing other people smoking the same brand (scores pre: 1.92, post: 1.65; p<0.001, V=261646) had also decreased at follow-up. There were no significant differences in the perceived taste and strength between brands, finding it hard to believe the cigarette brand they use is harmful, or feeling a connection with people who smoke the same brand.

**Table 5:** Comparison of tobacco-related perceptions at baseline (2019–2020) and follow-up (2021) in the Singapore Smokers Survey cohort, pre- and post-implementation of standardized tobacco packaging in Singapore (N=1873)

*Tobacco-related perceptions*	*Mean (SD)*			
	*Baseline*	*Follow-up*	*p^c^*	*V*
**Likert questions to assess perceived product appeal^a,b^**				
Aggregate product appeal score (**a–e**; max score: 25)	15.9 (3.3)***	14.3 (3.1)***		
a) Do you like the packaging of the tobacco products (cigarettes and other types of tobacco products) that you usually buy or use?	3.2 (1.1)***	2.7 (1.2) ***	<0.001	426738
b) How do you rate the tobacco products you currently use in terms of quality?	3.8 (0.7)***	3.6 (0.7)***	<0.001	230963
c) How do you rate the tobacco products you currently use in terms of satisfaction?	3.6 (0.7)***	3.5 (0.8)***	<0.001	198550
d) How do you rate the tobacco products you currently use in terms of value for money?	2.8 (1.1)***	2.6 (1.0)***	<0.001	318587
e) How do you rate the tobacco products you currently use in terms of appeal of the packaging?	2.5 (1.1)***	1.9 (1.1)***	<0.001	489051
**Likert questions related to other product attributes^b^**				
In your opinion, do you think that different cigarette brands taste different from each other?	2.3 (0.8)	2.3 (0.8)	0.711	152534
In your opinion, how different in strength are the varieties within a cigarette brand?	2.1 (0.8)	2.1 (0.8)	0.464	178030
In your opinion, are some cigarette brands more harmful than others?	0.6 (1.0)*	0.5 (0.9)*	<0.05	73209
Do you sometimes find it hard to believe that the cigarette brand you are using is harmful to your health?	2.8 (1.4)	2.7 (1.4)	0.166	232462
In the past month, how often have you noticed other people smoking the same brand of cigarettes as you?	1.9 (1.2)***	1.7 (1.2)***	<0.001	261646
Do you feel a connection with people who smoke the same brand as you?	2.6 (1.2)	2.6 (1.2)	0.127	190611
In the last one month, how often did you purposely cover up or conceal your pack of tobacco product(s), or put your tobacco product(s) in another container?	0.3 (0.8)	0.3 (0.8)	0.655	29029
In the last one month, to what extent – if at all – have the health warnings on the packs of tobacco products made you consider quitting smoking?	0.8 (1.0)*	0.9 (1.0)*	<0.05	116294
	***n (%)***	***n (%)***	***p^d^***	***χ^2^***
**In your opinion, do you think that some cigarette brands have more prestige (higher status) than others?^a^**			<0.001	25.9
Yes	1087 (58.0)**	1017 (54.3)**		
No	426 (22.7)***	543 (29.0)***		
Don't know/Not sure	360 (19.2)*	313 (16.7)*		
**When you see a pack of tobacco products that you usually buy or use, what do you usually notice first?^a^**			<0.001	43.4
The warning labels	385 (20.6)	420 (22.4)		
Other aspects such as branding	591 (31.6)***	435 (23.2)***		
Never really looked at the pack	806 (43.0)***	920 (49.1)***		
Don't know/Not sure	91 (4.9)	98 (5.2)		
**In the past month, have you asked for a pack of a tobacco product with a different health warning on it?^a^**			<0.001	14.3
Yes	131 (7.0)	130 (6.9)		
No	1549 (82.7)**	1611 (86.0)**		
Don't know/Not sure	193 (10.3)***	132 (7.0)***		

a Responses treated as categorical variables in our analysis.

b Responses scored on a five-point Likert scale and treated as continuous variables in our analysis. For Likert questions, maximum score is five unless indicated otherwise.

Statistically significant difference indicated by:

* p<0.05,

** p<0.01,

*** p<0.001.

c Wilcoxon signed rank test (continuous variables) or McNemar’s test (difference between proportions of categories for categorical variables).

d Bhapkar’s test.

In relation to the salience of health warnings, at follow-up, there was an increase in reporting that the health warnings made them consider quitting (scores pre: 0.81, post: 0.86; p<0.05, V=116294). When asked what they first notice on a tobacco pack, a higher proportion at follow-up indicated ‘never really looked at the pack’ (pre: 43.0%, post: 49.1%; p<0.001, χ^2^=43.4) and fewer indicated ‘other aspects such as branding’ (pre: 31.6%, post: 23.2%; p<0.001, χ^2^=43.4). There was no significant difference in purposely covering up or concealing the pack, and a higher proportion at follow-up reported not asking for a tobacco pack with a different health warning (pre: 82.7%, post: 86.0%; p<0.01, χ^2^=14.3) (Table 5).

## DISCUSSION

After the implementation of standardized packaging in Singapore, 11.7% of participants had quit smoking, more had cut down to non-daily smoking, and more reported intentions to quit compared at baseline. Evaluation studies from the United Kingdom and Australia have reported similar outcomes, with more people who smoke intending to quit^8,9,11^, foregoing cigarettes^8^, making quit attempts^11^, and making calls to the national quitline^16^, following the introduction of standardized packaging. Quit-related outcomes, including making a quit attempt, cutting down consumption, engaging with quit services and successfully quitting, are influenced by a wide array of factors such as the policy environment, availability of quit services, support from loved ones, personal readiness and the wider societal and cultural context^24^. Nevertheless, our findings suggest that, as in other countries, Singapore’s standardized packaging policy may have contributed to positive quit-related outcomes.

Those who quit after standardized packaging implementation were more likely to be younger (aged 18–24 years), smoking non-daily and intending to quit in the next 30 days or six months at baseline. Interestingly, we also found that those who had cut down, but not quit, were more likely to be Malay, smoking daily and with moderate or high dependence at baseline. This suggests that standardized packaging may sensitize some sub-groups to quitting or cutting down smoking more than others, and that those who did not successfully quit may have benefited from more targeted quit support during and after standardized packaging phase-in. The younger people smoking non-daily may have been more successful in quitting due to their lower dependence levels and pre-existing motivations to quit^25^. While such conclusions cannot be made based on the analyses presented in this study, further research in this area may be useful to assess the impact of standardized packaging on different sub-populations.

Singapore’s standardized packaging policy was associated with overall reductions in the perceived attractiveness of tobacco products, brand distinction, and perceptions that some brands are less harmful. Consistent with findings from Australia, Canada and England^6,7,9,13,14^, tobacco products were rated lower in terms of packaging, quality, satisfaction, value for money, and overall appeal, at follow-up. As in Australia and England^6,7,14^, the perceived differences between brands in terms of prestige and harmfulness were lower, and there was a decrease in noticing other people with the same brand at follow-up. Tobacco companies have long viewed the cigarette pack as an important marketing medium to communicate brand identity, reduced harm perceptions, and messages to appeal to youth and other vulnerable groups^26-28^. Our findings thus add to a growing evidence base which shows that standardized packaging, by eliminating the cigarette pack as a marketing medium, is an effective way to reduce the attractiveness of tobacco products as well as messages of reduced harm and brand identity.

As part of Singapore’s standardized packaging policy, graphic health warnings on packs were increased from 50% to 75% of the pack surface^2^; thus, the visibility of health warnings was increased by the absence of branding as well as their larger size. In Australia and the United Kingdom, standardized packaging measures increased avoidance behaviors such as covering up or concealing the pack or asking for a pack with a different health warning^4,5,7,8,11^, whereas in Singapore, we found no evidence of such changes. However, there was an increase in the proportion of those reporting that the health warnings motivated them to quit, consistent with findings from standardized packaging evaluations from Australia, the United Kingdom and France in which people who smoke reported increased concerns over their health^5,8,10^ and motivations to quit^7^ as a result of the graphic health warnings. Thus, our findings add to the evidence base which suggest that standardized packaging mandates are associated with increased salience of graphic health warnings.

At follow-up, more participants reported using flavored cigarettes. When standardized packaging was implemented in Singapore, the United Kingdom and Australia, tobacco companies intensified their marketing of flavored cigarettes, particularly flavor capsule variants, and diversified brand lines with novel flavors and filters^3,29-31^. These may deter quitting as they increase the appeal of tobacco products and some flavors, notably menthol, increase the addictiveness of nicotine^32^. While product diversification may reflect growth in the flavor capsule segment more generally^33^, tobacco companies use flavor capsule variants and other product innovations to target consumers in an increasingly regulated market^34,35^. The switching to flavored cigarettes, as observed in Singapore, may be a result of industry efforts to market flavored cigarettes prior to standardized packaging phase-in^3^, a general increase in their popularity, or both. It may also be due to variations in how the question to assess flavored cigarette use was asked in the baseline and follow-up questionnaires, as described above. To our knowledge, relatively few studies have examined changes in flavor preference in response to standardized packaging; there is a need for more monitoring of the tobacco industry’s strategies to undermine standardized packaging, as well as consumers’ responses.

At follow-up, more participants also reported using e-cigarettes. Although the sale, possession, and use of e-cigarettes are banned since 2017 in Singapore, cases of vaping have risen more generally following the re-opening of country borders after the COVID-19 pandemic^36^. While it is possible that standardized packaging of tobacco products may induce switching to alternatives that are packaged more attractively, such as e-cigarettes, the observed rise in vaping from baseline may also be a reflection of an increasing vaping trend in Singapore more generally.

### Limitations

Our study relied on self-reported data collected online or over the phone from participants who agreed to be recontacted for follow-up research, which may have influenced results due to selection bias or social desirability bias. Our sample was recruited via convenience methods and was not nationally representative. It included only Singaporean Citizens or Permanent Residents and did not capture changes among foreigners living in Singapore. Both surveys were completed during the COVID-19 pandemic which may have affected results. Notably, Singapore was in lockdown from 7 April 2020 to 1 June 2020 which coincided with some of the baseline data collection, and Singapore tightened its social distancing measures from 8 May 2021 to 29 March 2022 which coincided with follow-up data collection. Our data should also be interpreted within the limitations of the study’s non-causal design, possibility of residual confounding, and its measurements in the Singapore context, which may limit generalizability of findings to other countries.

## CONCLUSIONS

Our findings indicate that Singapore’s standardized packaging mandate is associated with positive quit-related outcomes, reduced attractiveness of tobacco products, brand distinction and the perceptions that some brands are less harmful, and increased effectiveness of graphic health warnings.

## Data Availability

The data supporting this research cannot be made available for privacy or other reasons.
